# Evaluation of the Characteristics of Sheep’s and Goat’s Ice Cream, Produced with UF Concentrated Second Cheese Whey and Different Starter Cultures

**DOI:** 10.3390/foods11244091

**Published:** 2022-12-17

**Authors:** Arona Pires, David Gomes, João Noronha, Olga Díaz, Angel Cobos, Carlos Dias Pereira

**Affiliations:** 1 Escola Superior Agrária, Politécnico de Coimbra, Bencanta, 3045-601 Coimbra, Portugal; 2 Departamento de Química Analítica, Nutrición y Bromatología, Área Tecnología de Alimentos, Facultad de Ciencias, Universidade de Santiago de Compostela, 27002 Lugo, Spain; 3 CERNAS—Centro de Estudos dos Recursos Naturais Ambiente e Sociedade, Bencanta, 3045-601 Coimbra, Portugal

**Keywords:** ovine, caprine, second cheese whey, ice cream, Kefir, yoghurt, probiotics

## Abstract

Second cheese whey (SCW) is the by-product resulting from the manufacture of whey cheeses. In the present work, sheep (S) and goat (G) SCW concentrated by ultrafiltration (UF) were used in the production of ice creams. Concentrated liquid SCW samples with inulin added as a prebiotic were fermented with yoghurt, kefir and probiotic commercial cultures before being frozen in a horizontal frozen yoghurt freezer. The physicochemical, microbiological and sensory properties of the products were evaluated over 120 days of frozen storage. The products presented significant differences regarding these properties, specifically the higher total solids and protein contents of sheep’s ice creams, which were higher compared to their goat ice cream counterparts. Sheep’s ice creams also presented higher hardness and complex viscosity, which increased with storage. These ice creams also presented higher overrun and lower meltdown rates. The color parameters of the ice creams showed significant differences between formulations resulting from storage time. In all cases, *Lactobacilli* sp. cell counts were higher than log 6 CFU/g at the first week of storage. In the case of sheep’s ice creams these values were maintained or increased until the 30th day, but decreased until the 60th day. *Lactococci* sp. counts surpassed log 7 CFU/g in all products, and these values were maintained until the end of storage, except in the case of G-Yoghurt and G-Kefir. Concerning the products containing probiotics, the sum of *Lactococci* sp. and *Lactobacilli* sp. counts was of the order log 8–9 CFU/g until the 60th day of storage, indicating that the probiotic characteristics of ice creams were maintained for at least 2 months. All products were well accepted by the consumer panel. Sheep’s SCW ice creams were better rated regarding aroma, taste and texture. However, only the ranking test was able to differentiate preferences among formulations.

## 1. Introduction

Cheese whey (CW) is the by-product resulting from the manufacturing of cheese. In some countries, CW is used to produce whey cheeses, such as *Ricotta* (Italy), *Requesón* (Spain) or *Requeijão* (Portugal). Particularly in the case of *Requesón* or *Requeijão*, the whey results from sheep’s or goat’s cheeses. However, not all the whey resulting from the manufacture of small ruminant’s cheeses is transformed into whey cheeses, with a large proportion being used as animal feed or directly discarded [1,2]. Even in the case of cow’s cheese whey, half of the global production is not valorized, despite its valuable nutritional composition [2]. Second cheese whey (SCW), also called deproteinized whey, is the by-product resulting from the production of whey cheeses. In previous works, we evaluated the characteristics and functional properties of sheep’s and goat’s whey and SCW. In addition, solutions for the valorization of these by-products were also proposed [3,4,5,6,7,8]. In some cases, the solutions envisaged their direct utilization after a concentration step performed by ultrafiltration (UF) [9,10,11]. Particularly in the case of SCW, only a few papers describe its characteristics and propose solutions for its treatment or valorization [12,13]. Other authors evaluated this material as a growth media to produce microalgae [14,15,16]. However, small scale dairy plants rarely apply such methodologies to valorize SCW. A possible solution can be the utilization of liquid SCW concentrated by ultrafiltration (UF) or nanofiltration (NF) as the main ingredient to produce fermented drinks or other dairy products, as is the case of ice cream. We recently evaluated the performance of sheep’s and goat’s whey in the production of frozen yoghurts [17].

Fermented dairy products are commonly produced in different parts of the world. Furthermore, the use of specific microbial cultures such as probiotic bacteria offers opportunities to develop innovative foods with extended shelf lives. Frozen dairy products can have properties of both yoghurt and ice cream, and can be the carriers of lactic acid bacteria, prebiotics and probiotics. The production of frozen yoghurts or ice creams with low fat or lactose levels and/or with added prebiotics and probiotics created new opportunities for the development of dairy foods with health-promoting properties. An increasing number of papers describe the formulations and characteristics of such products [18,19,20,21,22,23,24,25,26,27,28,29,30,31]. The production of ovine’s and caprine’s yoghurts and frozen yoghurts has also followed this trend. Several research works refer to the use of ewe’s [32,33,34,35,36,37] and goat’s [38,39,40,41,42,43,44,45,46,47,48,49,50] milks in the production of ice creams with functional properties. In addition, several authors reported the potential medicinal properties of small ruminant’s milks and dairy products [51,52,53].

Hence, the objective of this research was to manufacture ice creams using goat and sheep liquid SCW concentrates (LSCWCs) produced by UF, and to study their physicochemical, microbiological and sensory properties in order to evaluate the feasibility of the use of this by-product by the dairy industry. No reports were found in the literature regarding the manufacture of ice creams with goat and sheep liquid SCW concentrates.

## 2. Materials and Methods

### 2.1. Production of Liquid Whey Concentrates

Sheep and goat second cheese whey (SCW), supplied by external dairy companies, was transported to the pilot plant of the Escola Superior Agrária de Coimbra (Coimbra, Portugal), where it was processed. A total of 500 L of each type of SCW (ewe’s or goat’s) were subjected to ultrafiltration (UF) in a Proquiga Biotech SA pilot plant (A Coruña, Spain) equipped with a UF organic membrane (3838 PVDF/polysulfone) with an effective filtration area of 7 m^2^ and a 10 kDa cutoff, supplied by FipoBiotech, Spain. The process was carried out at 40–45 °C at a transmembrane pressure of 3 bar aiming at a volumetric concentration factor (VCF = Vol. feed/Vol. retentate) of 20, obtaining 25 L of concentrate. The concentrate was pasteurized (65 °C, 30 min) and then homogenized at 10 MPa using an APV Rannie™ homogenizer model Blue Top (Copenhagen, Denmark). Pasteurized and homogenized LSCWC were frozen at −25 °C until the moment they were used to produce ice creams.

### 2.2. Manufacture of Ice Creams

The sheep’s or goat’s LSCWC’s were thawed under refrigeration for 24 h. Subsequently, the samples were heated at 65 °C and homogenized at 10 MPa using APV Rannie™ model Blue Top (Copenhagen, Denmark). Afterwards, they were heated to 85 °C and passed through the homogenizer valve again. The concentrates were then cooled to 44 °C and divided into 6 L portions, to which the remaining ingredients were added in the following proportions (% *w*/*v*): sucrose (12%); inulin (5%), citric acid (0.4%) and xanthan gum (0.1%). The mix was allowed to mature for 12 h at 4 ± 2 °C. Finally, each mixture was inoculated with one of the following cultures:

1. Yoghurt culture: Yoflex™ (YF-L903, CHR Hansen, Hoersholm, Denmark) thermophilic yoghurt starter culture (*Lactobacillus delbrueckii* subsp. *bulgaricus* and *Streptococcus thermophilus*) at a concentration of 0.005% (*w*/*v*);
2. Kefir culture: Exact^TM^ Kefir 1 (CHR Hansen, Hoersholm, Denmark) mesophilic and thermophilic culture (*Debaryomyces hansenii*, *Lactococcus lactis* subsp. *cremoris*, *L. lactis* subsp. *lactis* biovar *diacetylactis*, *L. lactis* subsp. *lactis*, *Leuconostoc* and *Streptococcus thermophilus*) at a concentration of 0.005% (*w*/*v*);
3. A mixture of probiotic cultures: (*Bifidobacterium* BB-12, *Lactobacillus acidophilus* LA-5) and *Streptococcus thermophilus,* at a concentration of 0.005% (*w*/*v*). ABT-3^TM^ (CHR Hansen, Denmark).

The inoculated sheep or goat mixtures based on LSCWCs were placed in an incubation chamber (Jenogand, model Y—1000, Copenhagen, Denmark) at 43 °C, and the pH was monitored until it reached a value of 4.6. Fermentation was stopped by rapid cooling to 20 °C in less than 30 min. Afterwards, the fermented mixtures were placed in the refrigeration chamber at 4 ± 2 °C for 12 h.

For each formulation, 6 L batches of ice cream were produced and placed in a horizontal frozen yoghurt freezer for 40 min. Immediately after freezing, the temperature of ice creams was −6 ± 1 °C. After this process, the ice creams were packaged in 500 mL polypropylene boxes and stored in a freezer at −21 ± 1 °C for 120 days. All experiments were performed in triplicate.

### 2.3. Physico-Chemical Analysis

#### 2.3.1. Compositional Analysis

Dry matter was determined by drying the samples in a Schutzart DIN 40050-IP20 Memmert™ oven (Schwabach, Germany) according to the AOAC (1997) procedure for frozen yoghurt [54]. The ash content was determined by the incineration of dry samples in a Nabertherm™, model LE 4/11/R6 electric muffle furnace (Bremen, Germany) at 550 °C for 4 h, according to the AOAC method 935.42 [54]. The fat content was determined according to the AOAC method 952.06 [54]. The total N content was determined by the Kjeldahl method in the Digestion System 6 1007 Digester Tecator™ (Foss Analytical, Häganäs, Sweden) following the AOAC standard, and the conversion factor of 6.38 was used to calculate the percentage of protein [54]. All analyses were performed in triplicate.

#### 2.3.2. pH and Titratable Acidity

The pH was determined with a HI 9025 Hanna Instruments pH meter (Leighton Buzzard, UK) in order to monitor its evolution over fermentation, immediately after the production of the fermented products and on the 1st, 30th, 60th and 120th days of storage of ice creams. The pH meter was previously calibrated with 7.01 (HI5007) and 4.01 (HI5004) Hanna buffer solutions. The titratable acidity, expressed in g of lactic acid/L, was determined by means of titration using a 0.1 N NaOH solution according to the technique described in NP 701:1982 for yoghurts [55] and AOAC (1997) for frozen yoghurts [54]. For each sample, three determinations were made both for pH and for titratable acidity.

#### 2.3.3. Color Analysis

The color of the ice creams was determined with a Minolta Chroma Meter, model CR-200B colorimeter (Tokyo, Japan) calibrated with a white standard (CR-A47: Y = 94.7; x 0.313; y 0.3204). The following conditions were used: illuminant C, 1 cm diameter aperture, 10° standard observer. The color coordinates were measured in the CIEL*a*b* system.

Color difference (ΔEab*) was calculated as:

ΔEab* = [(L* − L*^0^)^2^ + (a* − a*^0^)^2^ + (b* − b*^0^)^2^]^1/2^ (1)

where L*^0^, a*^0^, and b*^0^ and L*, a*, and b* were the values measured for the samples under comparison. A matrix of ΔEab* values between products was constructed. Five measurements were taken for each sample.

#### 2.3.4. Rheological Analysis

The rheological properties of the different samples were determined in a Rheostress 1 rheometer (ThermoHaake™, ThermoFisher Scientific, Waltham, MA, USA) in oscillatory mode. The measurement system consisted of a cone and plate geometry, C60/Ti-0.052 mm (35 mm diameter and 1° angle). Stress sweep tests were conducted at 1 Hz to deter-mine the linear viscoelastic range of the yoghurts.

The complex viscosity (η*) of the products was evaluated in the range of 0.3 to 6.5 rad/s at 3 Pa. Three measurements were taken for each sample.

#### 2.3.5. Texture Analysis

A Stable Micro Systems texture analyzer, model TA.XT Express Enhanced (Godalming, UK), was used to evaluate the hardness of the frozen samples one day after production, and the results were calculated using Specific Expression PC software.

#### 2.3.6. Overrun

Overrun is the increase in the volume of the ice cream after freezing due to the incorporation of air. The method described by Skryplonek et al. [28] was followed, measuring the weight of the mixture and that of ice cream with the same volume. The determinations were done in triplicate.

Overrun [%] = (weight of mixture-weight of ice cream/weight of ice cream) × 100(2)

#### 2.3.7. Meltdown Rate

The meltdown rate was determined by the method described in Skryplonek et al. [28] by placing 30 g of ice cream on a stainless-steel screen with a 1 × 1 mm opening, located on top of a beaker. After 45 min at 20 ± 1 °C, the weight of the sample collected in the beaker was measured (*n* = 3). The meltdown rate was expressed as the percentage of the melted ice cream weight divided by the initial ice cream weight. The determinations were done in triplicate.

Meltdown [%] = (weight of melted ice cream/initial weight of ice cream) × 100 (3)

### 2.4. Microbiological Analysis

The microbial counts of lactic acid bacteria (LAB) of the genera *Lactobacillus* sp. and *Lactococci* sp. were determined after production and during the storage of frozen samples (1, 30, 60 and 120 days). *Lactobacilli* sp. and *Lactococci* sp. were enumerated on plates at 37 °C for 48 h on M17 agar (in aerobiosis) and on MRS agar (in anaerobiosis) (Biokar Diagnostics, France), respectively, according to ISO 7889, IDF 117 (2003) [56]. In this process, 1 mL of dilutions of 10^−5^, 10^−6^ and 10^−7^ were inoculated in triplicate along with two controls for each medium.

### 2.5. Sensory Analysis

Consumer preference tests were conducted with an untrained panel at the 7th, 30th, 60th and 120th days of storage. The hedonic test was used to determine the degree of acceptability of the products [57]. A category-type scale with an odd number (five) categories (1 = “I don’t like it at all” to 5 = “I like it very much”) was used. A neutral midpoint (neither like nor dislike) was included.

The six formulations were presented to panelists, who were asked to evaluate the characteristics of aroma, taste, color and texture using a hedonic scale of 1 to 5. Thirty-one consumers participated in the panel. The members of the panel were also asked to rank the samples according to their preference, from 1 (most preferred) to 6 (less preferred) [58].

### 2.6. Statistical Analysis

Prior to statistical analysis, normal distribution was tested using the Kolmogorov–Smirnov test. The one way ANOVA and the Tukey’s post hoc test were used to test and compare, the statistical significance of differences among means, respectively. Results from the sensory ranking test were evaluated by the chi square test. For all mean evaluations, a significance level of *p* < 0.05 was used (IBM SPSS Statistics version 19 for Windows; 2010; SPSS Inc., Chicago, IL, USA).

## 3. Results and Discussion

Table 1 presents the composition of goat and sheep LSCWCs. The values of dry matter, protein, fat and ashes in sheep LSCWC were higher than those observed in goat LSCWC. The pH values for sheep and goat liquid CSW concentrates were 6.51 and 6.36, respectively. The titratable acidity from goat LSCWC (1.02 ± 0.007%) was lower than that observed in sheep LSCWC (1.95 ± 0.07%).

**Figure 1:**
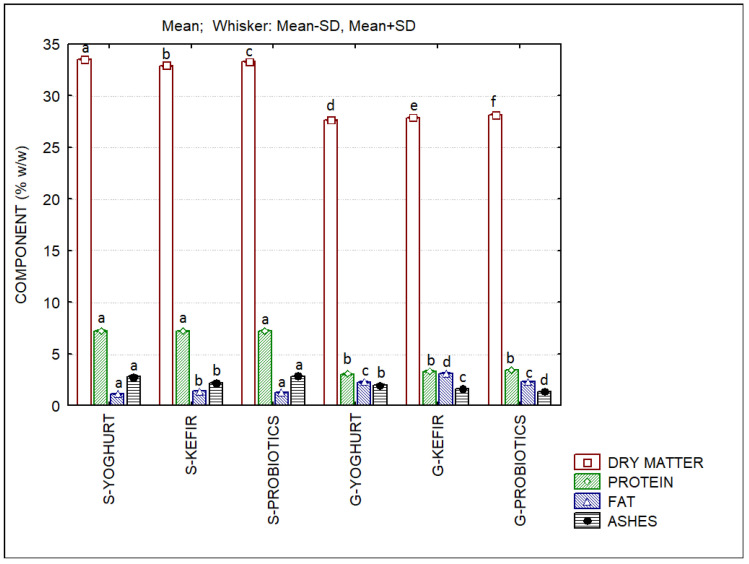
Chemical composition of sheep’s and goat’s second cheese whey ice creams produced with different starter cultures. Different letters of the same component indicate significant differences (*p* < 0.05) among ice creams.

The compositions of the ice creams are shown in Figure 1. Significant differences in dry matter were found among all samples. The protein content was higher in sheep’s SCW ice creams when compared to goat’s SCW ice creams. Sheep’s ice creams presented slightly lower fat contents and higher ash contents when compared to goat’s ice creams.

**Figure 2:**
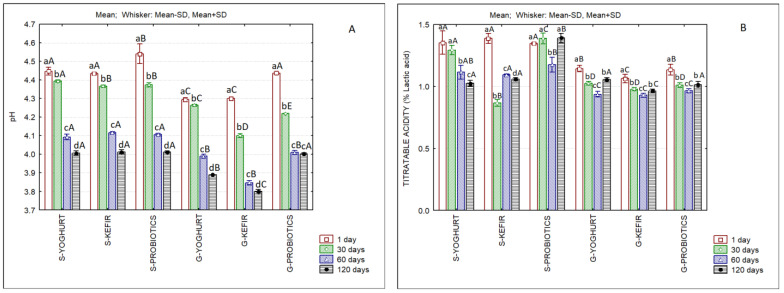
pH (**A**) and titratable acidity (**B**) of sheep’s and goat’s second cheese whey ice creams produced with different starter cultures over 120 days of frozen storage. Different lowercase letters in each type of ice cream indicate significant differences (*p* < 0.05) among storage days and different capital letters in each storage day indicate significant differences (*p* < 0.05) among ice creams.

Figure 2 shows the pH and titratable acidity of the different ice creams. The decrease in pH over storage time in all samples is clear. In the beginning of storage, goat’s SCW ice creams presented lower pH values when compared to their sheep’s counterparts, and this pattern was maintained until the end of storage, except for goat’s ice cream fermented with probiotics, whose pH is similar to all of the sheep’s ice creams at the first and 120th days of storage. However, the titratable acidity values do not reflect the evolution of pH, since sheep’s ice creams present higher values when compared to goat’s ice creams, and the opposite pattern was expected. The explanation for this finding can be attributed to the buffering effect exerted by whey proteins, as reported by other authors [59].

**Figure 3:**
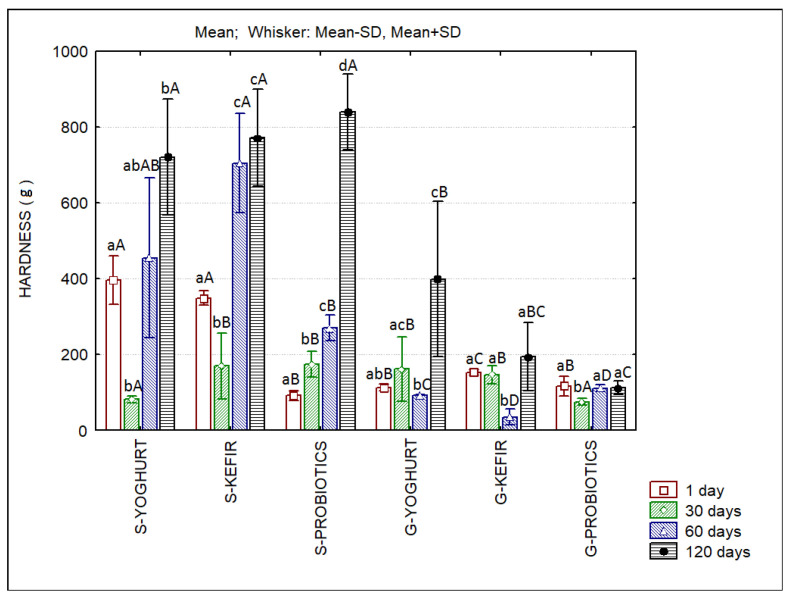
The hardness of sheep’s and goat’s second cheese whey ice creams produced with different starter cultures. Different lowercase letters in each type of ice cream indicate significant differences (*p* < 0.05) among storage days and different capital letters in each storage day indicate significant differences (*p* < 0.05) among ice creams.

**Figure 4:**
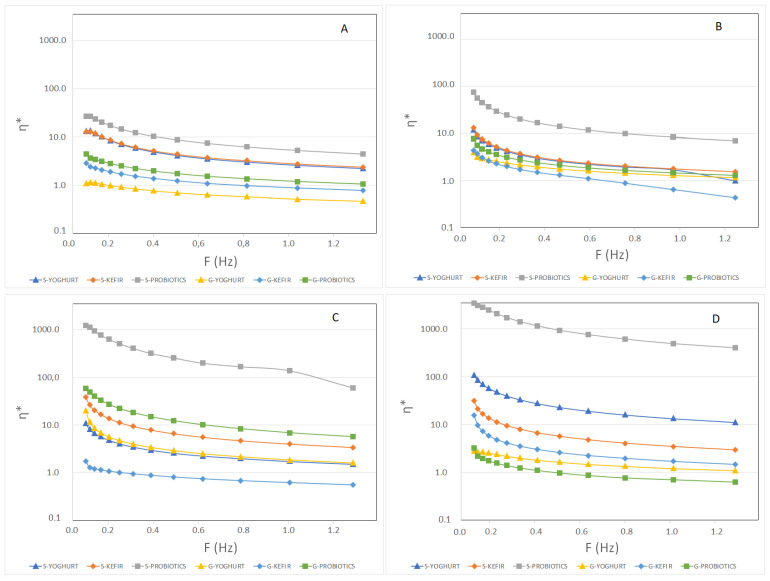
Complex viscosity (η*) of sheep’s and goat’s second cheese whey ice creams produced with different starter cultures over storage. 1 day (**A**); 30 days (**B**); 60 days (**C**); 120 days (**D**).

The hardness results of sheep’s and goat’s SCW ice creams produced with different starter cultures are presented in Figure 3. The texture results indicate higher values of hardness in the sheep’s ice creams with a tendency to increase over storage, exceptions being the cases of S-Yoghurt and S-Kefir at the 30th day of storage. At the end of storage, the hardness of sheep’s ice creams is clearly higher when compared to goat’s ice creams. The significantly higher amount of protein in sheep’s formulations, which more than doubled that of goat’s formulations, can explain these results. Small strain rheological tests (Figure 4) confirm the patterns observed with the texture analyzer. Sheep’s ice creams containing probiotics always presented the highest values for the complex viscosity (η*). S-Yoghurt, S-Kefir and S-Probiotics present higher values than their goat’s SCW ice cream counterparts. However, goat’s ice cream containing yoghurt (G-Yoghurt) presented η* values similar to that of its sheep’s counterpart at the 60th day of storage. At this moment, goat’s ice cream containing probiotics (G-Probiotics) presented higher values when compared with S-Yoghurt and S-Kefir.

**Figure 5:**
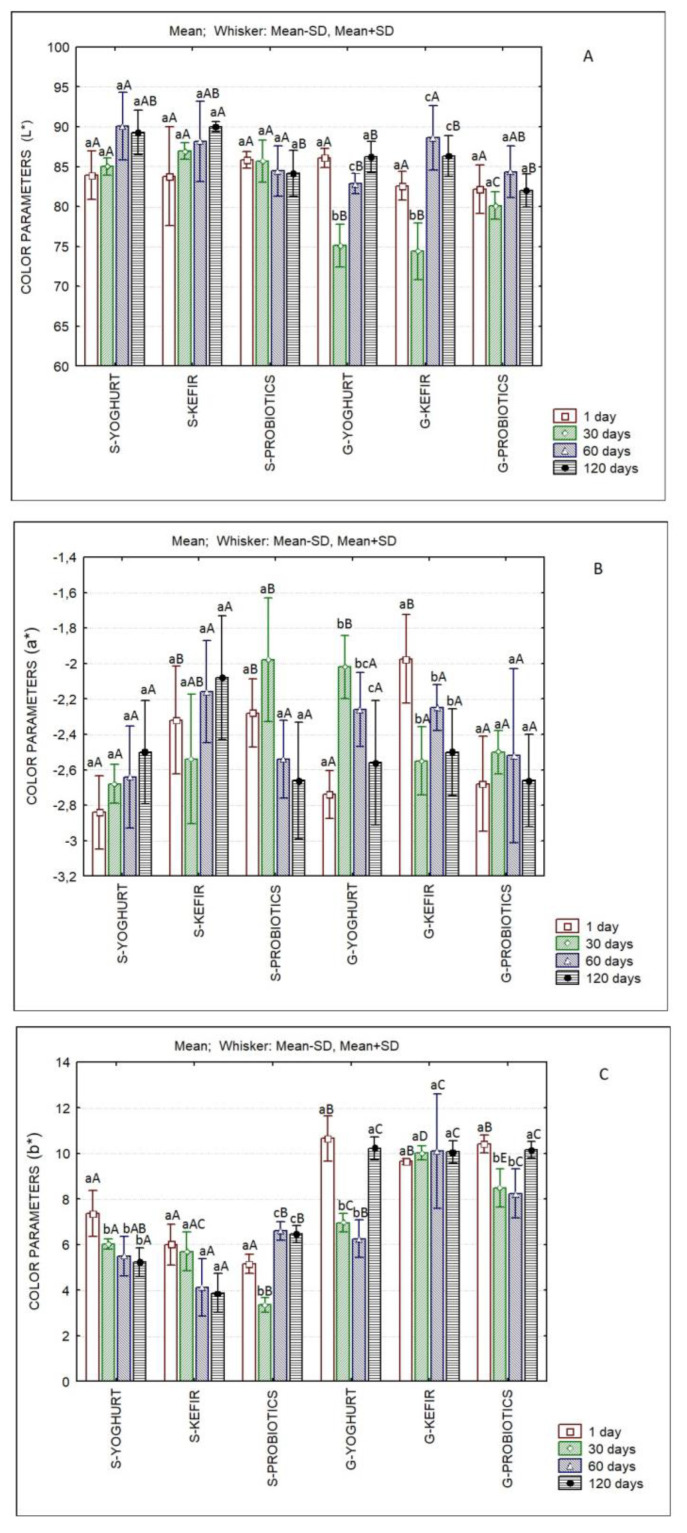
Evaluation of color parameters of sheep’s and goat’s second cheese whey ice creams produced with different starter cultures over storage. L* (**A**); a* (**B**); b* (**C**). Different lowercase letters in each type of ice cream indicate significant differences (*p* < 0.05) among storage days and different capital letters in each storage day indicate significant differences (*p* < 0.05) among ice creams.

Figure 5 presents the evolution of L* (lightness), a* (red-green axis) and b* (blue-yellow axis) of sheep’s and goat’s SCW ice creams produced with different starter cultures over storage. Significant differences were observed between products and over storage time. Table 2 and Table 3 describe the color differences (ΔEab*) of sheep’s and goat’s SCW ice creams produced with different starter cultures over storage and among products at the same storage day. With a few exceptions, ΔEab* values are higher than 1, indicating that a normal observer could detect color differences between ice creams [60]. It has to be pointed out that the exceptions were observed with respect to the same product at different storage times (e.g., the case of S-Probiotics between the 60th and 120th days of storage).

The overrun values (Table 4) are higher in sheep’s ice creams when compared to goat’s ice creams; however, in all cases, overrun values can be considered low when compared to data referred by other authors [17]. Regarding the meltdown rate, S-Yoghurt and S-Probiotics presented lower values, with the latter presenting the higher resistance to melt.

Few works refer to the use of dairy by-products in the manufacture of ice cream. De Meneses et al. [31] evaluated ice cream production with ricotta or cheese whey and buttermilk and the impact of such ingredients on quality parameters. The results indicated a significant reduction in fat content in ice creams with different by-products (40% on aver-age). The instrumental texture and color of the ice creams with ricotta and cheese whey and buttermilk showed a slight change, showing greater hardness and color intensity, which were not observed by consumers and did not affect the sensory tests. All ice cream samples had good overall liking values. Moschopoulou et al. [35] tested the employment of ovine and caprine whey protein concentrates (WPC) in the production of three ovine ice creams with 5% of lipids. The first ice cream had bovine skimmed milk powder (SMP) (35% protein), the second one contained ovine-caprine WPC with 65% protein (WPC65), and the third ice cream had ovine/caprine WPC with 80% protein (WPC80). The authors observed that the ice cream with SMP melted faster and showed higher overrun than ice creams with WPCs. Regarding hardness, the ice cream with SMP presented lower values when compared with the other samples. In color parameters, ice cream with SMP was significantly brighter, while ice creams with WPCs were more yellow. For the sensory properties, the flavor scores of ice cream with 65% protein WPC were similar to those of ice cream with SMP. Our results showed higher values for meltdown in goat ice creams. Regarding overrun, sheep ice creams presented higher values, most probably due to their higher protein content, which allowed for more air incorporation in the matrix. In hardness, sheep ice creams presented higher values and this can be attributable, once again, to their higher protein content. Both sheep and goat ice cream hardness tended to increase over storage. McGhee et al. [45] tested three goat milk ice creams manufactured using milk with three levels of lipids (3.64%, 2% and 0.71%) and studied their textural and sensory characteristics. The results showed very important increases in firmness and consistency in all three types of goat ice creams after 1 day of frozen storage. The increases of these properties were also important after 56 days of frozen storage. There was a slight decrease in overall acceptability of the three goat ice creams as the storage period increased, but they were acceptable after 8 weeks of frozen storage. Regarding hardness, we also observed an increase in hardness of all sheep ice creams and in goat´s ice cream containing yoghurt, but mainly after 120 days of storage.

**Figure 6:**
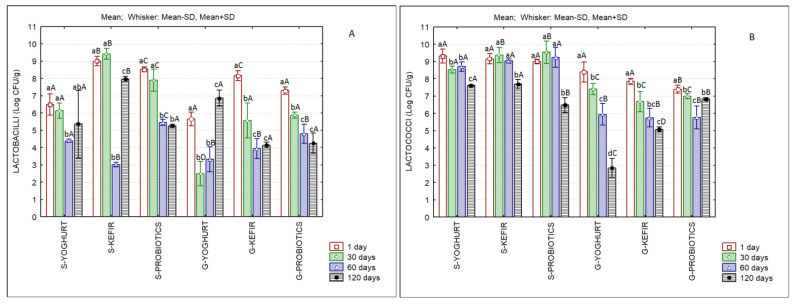
Evaluation of microbial counts of sheep’s and goat’s second cheese whey ice creams produced with different starter cultures over storage. *Lactobacilli* sp. (**A**); *Lactococci* sp. (**B**). Different lowercase letters in each type of ice cream indicate significant differences (*p* < 0.05) among storage days and different capital letters in each storage day indicate significant differences (*p* < 0.05) among ice creams.

Pimentel et al. [30] reviewed the technological and sensory aspects of probiotic ice creams and reported that these products showed satisfactory sensory properties as well as probiotic survival (>6 log CFU/g during storage and simulated gastrointestinal conditions). In our products, *Lactobacilli* sp. cell counts were higher than log 6 CFU/g at the first week of storage (Figure 6A). In the case of sheep’s ice creams, these values were maintained or increased until the 30th day, but decreased until the 60th day of frozen storage. However, S-Kefir presented counts of ca. log 8 CFU/g at the end of storage. *Lactococci* sp. counts surpassed log 7 CFU/g in all products, and these values were maintained until the end of storage, except in the case of G-Yoghurt and G-Kefir (Figure 6B). It must be stressed that, in the case of ice creams containing probiotic cultures (S-Probiotics and G-Probiotics), the sum of *Lactococci* sp. and *Lactobacilli* sp. counts was of the order of log 6–7 CFU/g until the 30th day of storage, indicating that the probiotic characteristics were maintained, at least for 1 month.

Other manuscripts reported high probiotic counts in ice creams. Senaka Ranadheera et al. [40] observed that the manufacturing process of probiotic ice cream originates a reduction in the viable cell numbers, but the products showed values of log 7–8 CFU/g over one year storage at −20 °C. Variations in body, texture and taste of the product were apparent after 12 weeks of storage. Camelo-Silva et al. [23] reported that concentrated milk protected Bifidobacterium BB-12 cells over storage and during in vitro gastrointestinal assays. For the ice cream manufactured with concentrated milk, a high bifidobacteria protective effect in the descending colon was observed, with probiotic viable cell counts and recovery rate values equal to 9.88 log CFU/g. Silva et al. [46] observed that the addition of the probiotic bacteria Bifidobacterium animalis subsp. lactis BLC1 in the manufacture of goat’s milk ice cream decreased the pH, but it had no effect on physicochemical properties, overrun or the melting behavior of this product. After 120 days of frozen storage, a high survival rate was detected. In addition, fine sensory scores were reported and adequate probiotic viability was maintained throughout the 120 days of frozen storage in this ice cream. Açu, et al. [38] produced ice cream with goat’s milk, milk powder, prebiotics and *Lactobacillus paracasei* subsp. paracasei and Bifidobacterium longum + Bifidobacterium bifidum combined culture (as probiotic cultures). Açu, et al. [42] also observed that these synbiotic ice creams maintained their probiotic properties during storage and had good sensory scores. de Paula et al. [48] manufactured goat milk ice creams with Lactobacillus rhamnosus or Lactobacillus paracasei as probiotic cultures, and these microorganisms had high viability levels during storage. The added probiotics maintained viability levels above 8 Log CFU/g during storage. Inulin added ice creams that showed lower overrun, while the hardness and meltdown rate increased in comparison with milk cream formulations. These products were well accepted by consumers. Shahein et al. [21] evaluated the microbiological and physicochemical characteristics of frozen yoghurt made with probiotic strains in combination with Jerusalem artichoke tubers powder (JATP) used as a fat and sugar replacer. Samples with JATP contained viable counts of Bifidobacterium bifidum BGN4 and Lactobacillus casei Lc-01 of 7 log CFU/g during 90 days of storage, as compared to the control sample. The addition of JATP also increased the acidity and enhanced the overrun melting resistance and viscosity of frozen yoghurts. The addition of JATP up to 10% also increased sensory attributes. Al-Shawi et al. [19] evaluated probi- 347 otic (*Lactobacillus acidophilus*), and synbiotic (*L. acidophilus* and inulin) ice creams and concluded that *L. acidophilus* counts were higher in synbiotic ice cream. Synbiotic ice cream received the highest overall acceptance scores. Sabet-Sarvestani et al. [20] produced synbiotic ice cream using Lactobacillus casei/Lactobacillus plantarum and fructooligosaccharides, and they observed a beneficial role of these prebiotics for the growth of probiotics. Kowalczyk et al. [34] observed that the survival of Bifidobacterium animalis ssp. lactis BB-12 in sheep’s milk ice cream at the 21st day of storage exceeded log 10 CFU/g. The overrun of the sheep’s milk ice cream was within the range of 78.50% to 80.41%. These values are clearly higher than the ones obtained by us. However, the differences could result from the fact that overrun values have a high dependence on the type of freezer used. Kowalczyk et al. [37] also observed that the freezing process reduced the population of probiotic bacteria cells in sheep´s milk ice cream with inulin from 0.8, 1.0 and 1.1 log CFU/g in products with *L. acidophilus* LA-5, *Lacticaseibacillus paracasei* L-26, and *Lacticaseibacillus casei* 431, respectively.

**Figure 7:**
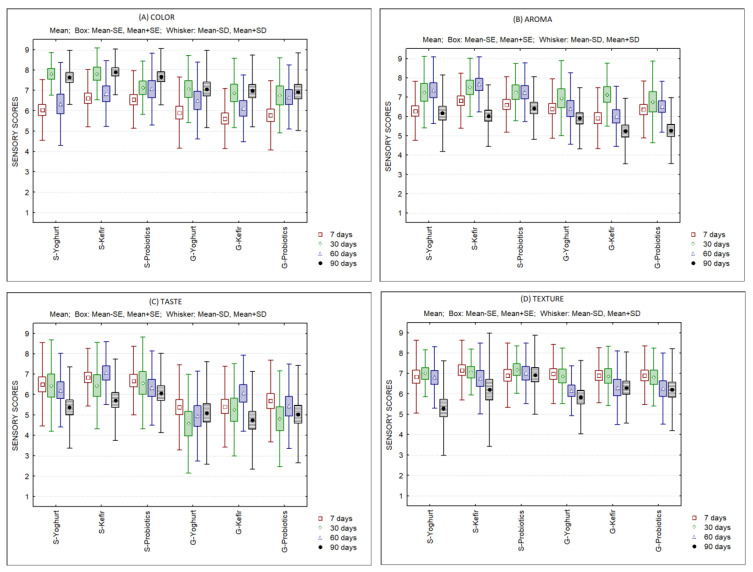
Sensory scores of sheep’s and goat’s second cheese whey ice creams produced with different starter cultures over 120 days of storage. Color (**A**); Aroma (**B**); Taste (**C**); Texture (**D**).

With regard to sensory quality, all products produced in the present work were well accepted by the consumer’s panel up to the 120th day of storage (Figure 7). Only the ranking test was able to differentiate preferences between products. Sheep’s ice creams were preferred in comparison to goat’s ice creams, specifically regarding taste. The ranking test indicated that at the 7th day of storage, the order of preference was as follows: S-Kefir, S-Probiotics, S-Yoghurt, G-Probiotics, G-Yoghurt and G-Kefir. However, no differences were observed between S-Yoghurt, G-Probiotics and G-Yoghurt. At the 30th day of storage, the order of preference was: S-Probiotics, S-Kefir, S-Yoghurt, G-Yoghurt, G-Kefir and G-Probiotics. Again, no differences were observed between S-Yoghurt, G-Yoghurt and G-Kefir. At the 60th day of storage, the order of preference was: S-Kefir, S-Yoghurt, S-Probiotics, G-Kefir, G-Probiotics and G-Yoghurt. At this time, sheep’s and goat’s ice creams were clearly separated with regard to consumer’s preference. At the end of storage, the order of preference was S-Kefir, S-Probiotics, G-Yoghurt, S-Yoghurt, G-Kefir, G-Probiotics.

## 4. Conclusions

The production of sheep’s or goat’s ice creams can represent an interesting opportunity for the valorization of SCW since it is infrequently reused. Whey and second cheese whey usually represent a problem to small and medium scale dairy processing units. These plants do not have adequate equipment to process SCW (i.e., concentration and drying equipment) and usually these by-products are offered for livestock feeding or are discarded into the public sewage system. The use of ultrafiltration, or even nanofiltration, to concentrate SCW originates liquid second cheese whey concentrates (LSCWCs) which can be directly used in the development of novel dairy products. This solution allows for the reduction in negative environmental effects resulting from the direct discharge of SCW. Furthermore, the addition of prebiotics and the production of fermented products containing probiotic bacteria increases the nutritional value of LSCWC’s and their market potential. The results of the present work have practical applications in small and medium scale dairy plants by reducing their environmental impact and improving their profitability via value-added products, which is crucial for sustainable rural development.

## Figures and Tables

**Table 1:** Composition of sheep’s and goat’s liquid second cheese whey concentrates (LSWCs).

LSCWC	Dry Matter (%)	Protein (%)	Fat (%)	Ashes (%)
Sheep	20.84 ± 0.75	9.58 ± 0.27	2.10 ± 0.001	1.65 ± 0.01
Goat	12.38 ± 0.01	2.96 ± 0.003	1.60 ± 0.001	1.57 ± 0.004

**Table 2:** Color differences (ΔEab*) of sheep’s and goat’s second cheese whey ice creams produced with different starter cultures over storage.

Products	1 d. vs. 30 d.	1 d. vs. 60 d.	1 d. vs. 120 d.	30 d. vs. 60 d.	30 d. vs. 120 d.	60 d. vs. 120 d.
S-Yoghurt	3.6	35.4	24.1	22.2	13.3	1.9
S-Kefir	24.8	45.9	35.3	11.6	8.1	12.3
S-Probiotics	4.8	5.6	5.6	13.5	10.7	0.8
G-Yoghurt	72.7	17.9	1.0	34.1	72.8	18.0
G-Kefir	37.2	32.5	11.2	119.3	80.4	16.8
G-Probiotics	8.2	17.3	0.5	17.8	13.8	13.8

**Table 3:** Color differences (ΔEab*) of sheep’s and goat’s second cheese whey ice creams produced with different starter cultures. Comparison between products at the same storage time.

Product Comparison	1 Day	30 Days	60 Days	120 Days
S-Kefir vs. S-Yoghurt	9.8	3.6	13.7	4.2
S-Probiotics vs. S-Yoghurt	9.9	5.5	24.8	17.1
S-Probiotics vs. S-Kefir	22.2	7.3	14.4	23.7
G-Yoghurt vs. S-Yoghurt	14.3	54.2	33.7	23.2
G -Kefir vs. S-Yoghurt	4.1	65.8	35.9	20.9
G-Probiotics vs. S-Yoghurt	7.7	18.0	32.6	46.5
G-Kefir vs. S-Kefir	19.7	96.9	28.1	27.7
G-Probiotics vs. S-Kefir	22.8	30.2	23.5	53.2
G-Probiotics vs. S-Probiotics	26.0	31.7	8.9	11.3
G-Yoghurt vs. G-Kefir	9.6	13.7	34.5	5.4
G-Yoghurt vs. G-Probiotics	29.8	30.0	5.7	22.5
G-Kefir vs. G-Probiotics	2.3	18.4	21.9	15.1

**Table 4:** Overrun and meltdown rate of sheep’s and goat’s second cheese whey ice creams produced with different starter cultures. Different letters in each column indicate significant differences (*p* < 0.05) between ice creams.

Products	Overrun (%)	Meltdown Rate (%)
S-Yoghurt	18.34 ± 3.12 ^a^	43.39 ± 2.16 ^a^
S-Kefir	20.93 ± 5.30 ^ab^	61.10 ± 6.95 ^b^
S-Probiotics	30.92 ± 4.64 ^b^	21.45 ± 2.22 ^c^
G-Yoghurt	13.53 ± 1.06 ^c^	73.94 ± 1.28 ^b^
G-Kefir	13.87 ± 9.79 ^c^	71.08 ± 2.26 ^b^
G-Probiotics	12.51 ± 7.07 ^c^	63.37 ± 6.32 ^b^

## Data Availability

Data is contained within the article.
